# Impact of 12-week exercise program on biomarkers of gut barrier integrity in patients with coronary artery disease

**DOI:** 10.1371/journal.pone.0260165

**Published:** 2021-11-19

**Authors:** Vivian Feng, Kritleen K. Bawa, Susan Marzolini, Alex Kiss, Paul Oh, Nathan Herrmann, Krista L. Lanctôt, Damien Gallagher

**Affiliations:** 1 Department of Pharmacology & Toxicology, University of Toronto, Toronto, Ontario, Canada; 2 Hurvitz Brain Sciences Program, Sunnybrook Research Institute, Toronto, Ontario, Canada; 3 Neuropsychopharmacology Research Group, Sunnybrook Research Institute, Toronto, Ontario, Canada; 4 Heart and Stroke Foundation Canadian Partnership for Stroke Recovery, Sunnybrook Research Institute, Toronto, Ontario, Canada; 5 KITE Toronto Rehabilitation Institute, University Health Network, East York, Ontario, Canada; 6 ICES, Toronto, Ontario, Canada; 7 Institute of Health Policy, Management and Evaluation, University of Toronto, Toronto, Ontario, Canada; 8 Department of Psychiatry, Faculty of Medicine, University of Toronto, Toronto, Ontario, Canada; Prince Sattam Bin Abdulaziz University, College of Applied Medical Sciences, SAUDI ARABIA

## Abstract

**Introduction:**

Breakdown of gut barrier integrity has been associated with inflammatory activation and is implicated in the etiology of several chronic medical conditions. Acute exercise is known to increase gut barrier permeability but the impact of chronic exercise is not clear. Most studies to date have examined how acute exercise impacts gut barrier integrity in healthy adults, while few studies have examined the impact of chronic exercise in older adults with comorbidities. We aim to investigate the impact of a 12-week program of aerobic and resistance training on biomarkers of gut barrier integrity in a sample of older adults with coronary artery disease.

**Methods:**

Participants were adults with coronary artery disease undergoing a moderate-intensity 12-week cardiac rehabilitation exercise program. Fasting blood samples were taken at baseline and study termination. Serum levels of biomarkers of gut barrier integrity (zonulin and fatty acid-binding protein 2 (FABP2)) were measured by ELISA. Cardiorespiratory fitness was assessed by peak oxygen uptake (VO_2peak_) at study start & completion. Data analyses were performed using SPSS software version 24.0.

**Results:**

Among study participants (n = 41, 70% male, age = 62.7± 9.35) we found a significant negative association between baseline FABP2 levels and baseline VO_2peak_ in a multiple linear regression model adjusting for covariates (*B* = -0.3, p = 0.009). Over the course of the exercise program an increase in VO_2peak_ (≥ 5 mL/kg/min) was independently associated with a relative decrease in FABP2 (*B* = -0.45, p = 0.018) after controlling for medical covariates.

**Conclusion:**

Our findings indicate that an increase in cardiorespiratory fitness during a 12-week exercise program resulted in a relative improvement in a biomarker of gut barrier integrity. This indicates a potential mechanism by which longer term exercise may improve gut barrier integrity.

## 1. Introduction

Breakdown of the gut barrier has been implicated in the etiology of several chronic medical conditions associated with inflammatory activation including diabetes and cardiovascular disease [1]. Leakage of bacterial products such as lipopolysaccharide (LPS), a component of the bacterial cell wall and potent inflammatory stimulus, into the systemic circulation can perpetuate inflammatory activation. LPS activates immune cells via LPS-binding protein (LBP)/soluble CD14 (sCD14)/toll-like receptor-4 (TRL4) signalling and has been implicated in the etiology of atherosclerosis among other inflammatory conditions [1, 2]. Tight junctions between intestinal epithelial cells control flow of contents from the intestinal lumen to the systemic circulation and are critical to integrity of the gut barrier. Intestinal infections or inflammatory bowel disease can disrupt gut barrier integrity causing elevations of these biomarkers but lifestyle factors such as physical activity and diet are also known to be important [3].

Exercise is known to have beneficial effects in chronic medical conditions associated with inflammatory activation and increase gut barrier permeability. Physical exercise, such as marathon running has been known to increase gut barrier permeability and cause severe gastrointestinal disturbances [4] but even shorter acute bouts of exercise such as treadmill training for 60 minutes have similarly been associated with increased permeability and inflammatory activation [5]. To date, most studies have focused upon the effects of acute exercise in younger healthy adults and relatively few studies have examined the impact of chronic exercise in older adult populations with comorbid medical conditions. There is some emerging evidence that chronic moderate intensity exercise may improve gut barrier integrity. One recent study in adults with type 2 diabetes found that a six-month exercise program improved gut barrier integrity and reduced inflammatory activation which was closely correlated with improvement in gut barrier biomarkers [6]. In addition to some uncertainty regarding the duration and intensity of exercise necessary to improve gut barrier integrity, little is known about how the response to exercise or changes in cardiorespiratory fitness impact gut barrier integrity. Cardiorespiratory fitness is known to increase some protective species of gut microbiota [7] but its impact on biomarkers of gut barrier permeability is not clear.

We therefore undertook to investigate the impact of a 12-week exercise training program on serum biomarkers of gut barrier integrity in adults with coronary artery disease (CAD). We further wished to evaluate whether changes in cardiorespiratory fitness would be more closely associated with changes in these biomarkers than just engaging in exercise. Serum zonulin and intestinal fatty acid binding protein (FABP2) are two blood-based biomarkers associated with increased intestinal permeability. Zonulin is a protein that induces disassembly of tight junctions between cells of the duodenum and small intestine, resulting in increased permeability [8]. FABP2, is a protein found in the cytoplasm of enterocytes of the small intestine with elevated levels indicating enterocyte damage [9]. We hypothesized that a 12 week exercise program would be associated with a significant reduction in serum zonulin and FABP2. We further hypothesized that change in cardiorespiratory fitness would be more closely associated with beneficial effects. Lastly, we hypothesized that serum inflammatory markers would decrease with exercise and would be associated with biomarkers of gut barrier integrity.

## 2. Methods

### 2.1 Participants

Eligible patients were those that provided written informed consent with evidence of CAD. Data for this study were collected from patients with CAD undergoing cardiac rehabilitation who had been recruited for a randomized controlled trial testing omega 3 polyunsaturated fatty acid (n-3 PUFA) supplementation compared with placebo. History of CAD was defined as any myocardial infarction or coronary artery bypass graft or percutaneous transluminal coronary angioplasty or at least a 50% stenosis in 1 or more major coronary artery. English speaking participants aged between 45 and 85 were included. Subjects were excluded if they had the following diagnoses: neurodegenerative disease, unstable angina, substance abuse, women of childbearing potential, allergy or hypersensitivity to fish, contraindications to soybean/corn oil, a pre-existing bleeding disorder or cognitive impairment (Mini Mental Status Examination score <24). Antidepressant use was permitted if used at a stable dose for at least 3 months before the trial.

### 2.2 Trial design

Study participants were recruited at baseline from 12-week cardiac rehabilitation programs in Toronto, Ontario—University Health Network at Toronto Rehab or Trillium Health Partners. Patients were randomized to the double-blind phase in which they received either n-3 PUFA supplements or placebo for 12 weeks. Randomization was done in a 1:1 ratio using a block randomization code that was computer-generated at Sunnybrook Hospital and all research personnel remained blind to treatment allocation.

### 2.3 Omega 3 intervention

This was 3 capsules (3x1g) fish oil-derived concentrated ethyl esters, providing 1.9 g ω-3 FAs daily (1.2 g EPA and 0.6 g DHA, with 0.1 g other ω-3 FAs). A matching placebo of 3 capsules (3x1g) of 50/50 soybean/corn oil blend containing less than 0.12 g ω-3 FAs with negligible EPA and DHA was used. This trial and secondary analyses of data were approved by the research ethics boards of Sunnybrook Research Institute and University Health Network. More details about the trial design have been published elsewhere [10].

### 2.4 Exercise intervention

Exercise at each site consisted of supervised in-class aerobic and resistance training once weekly with additional at-home sessions 4 days per week for 12 weeks (11). Exercise prescriptions were based on cardiopulmonary assessments developed by the cardiac rehabilitation program. A typical supervised class is led by a half an hour lecture, followed by 15 minutes of warm-up and stretching exercises prior to completing their prescribed exercise with adequate staff supervision. The exercise training was for approximately 60 minutes and of moderate intensity. Initial prescriptions had an intensity equivalent to 60% of VO2 peak and prescriptions were advanced biweekly, increasing intensity to a maximum of 80% of VO2peak where exercise training was then maintained thereafter. High-risk patients, including those with chronic heart failure, cardiac transplantation, complex ventricular ectopy, symptomatic ST-segment depression, or atrial fibrillation may be scheduled initially for up to 5 weekly supervised sessions, reducing to one or two when warranted [11]. Peak volume of oxygen utilized or VO_2peak_ was measured at study intake and completion. Cardiorespiratory fitness was assessed by a cycle ergometer (Ergoline 800 EL) symptom-limited graded exercise test. Workload was increased by 16.7 watts every minute and breath-by-breath gas samples were collected and averaged over a 20 second period via a calibrated metabolic cart as described elsewhere [12]. The peak volume of oxygen uptake (VO_2peak_) was recorded and normalized for body mass (reported in mL/kg/min). The VO_2peak_ thus obtained represents a measure of ventilatory capacity at peak effort and it is a highly reliable and reproducible measure of cardiorespiratory fitness [13]. Personalized dietary guidance was not provided within the exercise intervention; however, the participants were provided with educational information on healthy diet choices.

### 2.5 Biomarkers of gut barrier integrity

Fasting blood samples were taken at baseline and at the termination of the study. Serum samples were stored at -80C until analysis. Serum zonulin was assayed using an ELISA kit (ALPCO, Salem, NH) and serum FABP2 by sandwich ELISA (R&D Systems Quantikine kit). Serum biomarker samples that were undetectable by ELISA were removed from the analysis. Retrospective samples were not anonymized and have been approved by Sunnybrook Research Institute ethics committee to be used for the purpose of this research.

### 2.6 Medical conditions

Details of study participant demographics, past medical history, and concurrent medications were collected. Comorbid medical conditions diagnosed by a physician were collected as part of a structured interview during which participants were asked about any other chronic dermatologic, gastrointestinal, cardiorespiratory, musculoskeletal, or neurologic conditions.

### 2.7 Statistical analyses

Baseline characteristics of all participants were represented as mean and standard deviation for continuous variables or number and percentage for categorical ones. A paired-samples two-tailed t-test was performed to assess the changes in gut integrity and inflammatory biomarkers following exercise training. ANCOVAs were performed to assess the difference in gut integrity biomarker concentrations between the treatment and placebo groups. Multiple linear regression models with a significance level of 0.05 were used to test the cross-sectional and longitudinal associations of gut integrity marker concentrations with VO_2peak_ while adjusting for potential confounders. Biomarker samples were tested for normality using the Shapiro-Wilk Test, skewness and kurtosis values. Variables were assessed for multicollinearity using tolerance values greater than 1. All analyses were performed using SPSS statistical software (version 24.0, IBM, NY, USA).

### 2.8 Outcome measures and other covariates

Zonulin and FABP2 were selected as measures of gut integrity, and hence were the outcome measures of interest in this study. Zonulin is a protein that induces disassembly of tight junctions between cells of the duodenum and small intestine, resulting in increased permeability. FABP2, is a protein found in the cytoplasm of enterocytes of the small intestine with elevated levels indicating enterocyte damage. Other covariates of interest included: cardiorespiratory fitness was measured by VO_2peak_ and assessed using a cycle ergometer. VO_2peak_ represents a measure of ventilatory capacity at peak effort and it is a highly reliable and reproducible measure of cardiorespiratory fitness. Serum markers of inflammatory activation: C-Reactive protein (CRP), Interleukin-6 (IL-6), and tumor necrosis factor (TNF) in the plasma from fasting blood at baseline and week 12 visits.

## 3. Results

### 3.1 Participant characteristics

A total of 41 baseline and termination values for zonulin and 36 baseline and termination FABP2 were available. Differences in number of FABP2 and zonulin values were due to having insufficient serum for a small number of subjects and participants without values were excluded from the relevant analysis. Of all 41 study participants, 20 were randomized to treatment taking 1.9 g ω-3 FAs daily while 21 were in the placebo group. All study participants took part in the exercise program. Baseline characteristics of the included participants are presented in Table 1.

**Table 1:** Baseline characteristics of all participants.

Characteristic	Mean ± SD or n (%)
**Age (years)**	62.66 ± 9.36
**Years Smoked**	16.37 ± 18.02
**Alcoholic Drinks/week**	3.97 ± 6.99
**VO_2peak_ (mL/kg/min)**	18.35 ± 4.56
**BMI kg/m^2^**	28.67 ± 4.78
**Male percent**	29 (70.7)
**Ethnicity—Caucasian**	30 (73.2)
**History of depression**	18 (43.9)
**NSAID (includes ASA)**	33 (80.5)
**Proton-Pump Inhibitor**	11 (26.8)
**Beta Blocker**	30 (73.2)
**Antidepressant use**	4 (9.8)
**Metformin use**	33 (80.5)
**Diabetes**	10 (24.4)
**Hypertension**	27 (65.9)
**Hypercholesterolemia**	34 (82.9)
**Omega 3 treatment**	20 (48.8)
**FABP2 (pg/mL)**	540.96 ± 318.39
**Zonulin (ng/mL)**	2.42 ± 0.35
**CRP (mg/L)**	0.023 ± 0.019
**TNF (pg/mL)**	6.59 ± 3.64
**IL-6 (pg/mL)**	3.14 ± 1.91

### 3.2 Associations with biomarkers of gut barrier integrity at baseline

In bivariate regression analyses the only demographic & clinical predictors significantly associated with FABP2 in all study participants at baseline was VO_2peak_ (F(1, 34) = 5.169, SE = 0.007, *B* = -0.363, p = 0.029, 95% CI = 2.697, 3.250) and metformin use (F(1, 34) = 5.299), SE = 0.082, *B* = 0.325, p = 0.028, 95% CI = 2.554, 2.710). The association between baseline VO_2peak_ and FABP2 remained significant following adjustment for metformin use in multiple linear regression (F(2, 33) = 5.442, SE = 0.07, B = -0.337, p = 0.009, 95% CI = 2.646, 3.180).Zonulin was not associated with any demographic or clinical factors at baseline. No significant associations between baseline VO2peak and zonulin were found in a linear regression model.

### 3.3 Predictors of change in biomarkers of gut barrier integrity during intervention

Levels of zonulin (df = 40, t = -1.276, SE = 0.04, p = 0.209, 95% CI = -0.03, 0.12) and FABP2 (df = 35, t = 0.201, SE = 0.032, p = 0.842, 95% CI = -0.06, 0.07) did not change significantly between baseline and follow up among all study participants. In an ANCOVA, randomization to omega 3 supplementation was not significantly associated with change in zonulin (F(1,36) = 1.197, adjusted R squared = 0.005, p = 0.281) or FABP2 levels (F(1, 36), adjusted R squared = -0.028, p = 0.814).

### 3.4 Cardiorespiratory fitness

During the study intervention there was a mean increase of 3.31 ±4.76 mL/kg/min in VO_2peak_ (t = 3.87, p = 0.001) among all study participants. The top quartile of study participants demonstrated an increase of VO_2peak_ (≥5 mL/kg/min). While FABP2 did not decline significantly in the overall sample, a VO_2peak_ improvement (≥5 mL/kg/min) was significantly associated with a relative reduction in FABP2 in a regression analysis with change in FABP2 (log transformed) as the outcome variable (*B* = 0.530, p = 0.013). Participants who demonstrated an improvement in VO_2peak_ of ≥ 5 mL/kg/min had a significantly lower mean termination FABP2 level of 378.61 (SD = 86.82) ng/ml than those who did not with a mean termination level of 607.02 (SD = 284.47) ng/ml (t = 3.191, p = 0.004). FABP2 baseline and termination values according to change in VO_2peak_ are displayed in Table 2. In bivariate regression analyses testing all variables from Table 1, the only other clinical variable significantly associated with change in FABP2, was hypercholesterolemia (*B* = 0.448, p = 0.006). The association between an improvement in VO_2peak_ (≥ 5 mL/kg/min) and reduction in FABP2 remained significant following adjustment for hypercholesterolemia in multivariable linear regression analysis with FABP2 as the dependent variable (*B* = -0.446, p = 0.018). Change in VO_2peak_ was not found to be significantly associated with changes in zonulin levels (*B* = 0.034, p = 0.857).

**Table 2:** Baseline and termination values of FABP2 for each quartile of VO_2peak_ change.

VO_2peak_ change quartile (range)	FABP2 at baseline (pg/mL) (mean ± SD)	FABP2 at termination (pg/mL) (mean ± SD)
Quartile 1 (-3.4 to 0.2)	479 ± 172	532.80 ± 184.76
Quartile 2 (0.4 to 1.4)	385.91 ± 210.40	489.82 ± 208.19
Quartile 3 (2.2 to 4.8)	612.13 ± 344.11	750.59 ± 352.49
Quartile 4 (5 to 19.1)	456.22 ± 134.89	378.61 ± 86.82

### 3.5 Inflammatory biomarkers

Inflammatory biomarker (CRP, TNF, and IL-6) values were skewed and were log transformed to normalize distribution. There were no significant differences between baseline and follow-up values for all inflammatory biomarkers using paired-samples t-tests CRP (df = 33, t = -0.84, SE = 0.06, p = 0.40, 95% CI = -0.16, 0.06), TNF (df = 36, t = -1.17, SE = 0.03, p = 0.25, 95% CI = -0.08, 0.02), IL-6 (df = 36, t = -0.84, SE = 0.04, p = 0.41, 95% CI = -0.12, 0.05). In regression analyses none of the inflammatory markers were significantly associated with baseline values of FABP2, Zonulin or change in Zonulin, FABP2 values during the study.

## 4. Discussion

In this study, we investigated how a 12-week exercise intervention impacted biomarkers of gut barrier integrity, zonulin and FABP2, in patients with CAD. While we did not find that participation in the exercise program per se impacted gut biomarkers we found that FABP2 had a significant negative association with VO_2peak_ at baseline and that an increase (≥ 5 mL/kg/min) in VO_2peak_ was significantly associated with a relative reduction in FABP2 compared to those who did not demonstrate an equivalent improvement in cardiorespiratory fitness. We did not find a significant association between zonulin and VO2_peak_. Omega 3 supplementation was not significantly associated with a change in zonulin or FABP2. In this analysis, inflammatory markers were not impacted by exercise and were not associated with changes in zonulin or FABP2.

Our main finding that FABP2 was negatively correlated with cardiorespiratory fitness at baseline and change in FABP2 during the intervention is consistent with a small but increasing body of evidence indicating that chronic moderate-intensity exercise may be associated with improvement of gut barrier integrity. While shorter episodes of exercise have been associated with an increase in FABP2 [14, 15] there is some emerging evidence to indicate that chronic exercise may be associated with longer term improvements in gut barrier integrity [16]. Importantly, in this analysis, we demonstrated that an improvement in cardiorespiratory fitness is more closely related to beneficial impacts on gut health. This is consistent with one other recently published analysis in adults with CAD which found an inverse relationship between cardiorespiratory fitness and biomarkers of gut permeability [17]. Possible mechanisms underlying increased intestinal permeability during acute exercise include a decrease in splanchnic blood flow as blood is diverted away from the gut to prioritize other organs potentially resulting in hypoxia and reperfusion injury of gut epithelial cells following acute exercise [18]. In contrast, there are physiologic adaptations with habitual exercise such that the body may mount a reduced sympathetic response allowing for improved splanchnic blood flow during exercise [19]. Chronic exercise is also associated with an increase in gut microbial populations that have been associated with improved gut barrier integrity. Specific bacterial populations increase during exercise and produce more short chain fatty acids such as butyrate that have been linked with improved gut barrier integrity [16, 20]. Lastly chronic exercise in previous studies has been shown to reduce pro-inflammatory cytokines that have been associated with increased gut permeability and increase antioxidant defences potentially improving resistance to oxidative stress during exercise [21]. We note that in this analysis we did not find a significant reduction in inflammatory biomarkers with exercise likely reflecting already low baseline levels in this sample of motivated patients receiving treatment in a specialty clinic.

We did not find a significant association between cardiorespiratory fitness and zonulin at baseline or during follow-up. Serum zonulin levels have previously been reported to increase following acute exercise [22] while one longer exercise intervention over 6 months in patients with type 2 diabetes demonstrated a reduction in zonulin indicating improved gut barrier integrity [6]. The lack of association here may reflect the small number of subjects under study or the relatively shorter duration (12 weeks) of this exercise intervention compared to the other 6-month study in this area. It is also possible that zonulin is less sensitive to changes in cardiorespiratory fitness as compared to FABP2 and it has previously been noted that variability in zonulin assays may have contributed to conflicting findings in previous analyses [23]. We note that a small proportion of participants (19%) did not exhibit an improvement in VO2peak which may reflect lower adherence or reduced responsivity to exercise given individual variability in cardiorespiratory adjustment to exercise [24].

Omega 3 fatty acids are known to promote the growth of bacterial species associated with enhanced gut barrier integrity through increased production of protective bacterial products such as the short chain fatty acid, butyrate [25]. We therefore conducted a sub-analysis to determine if randomization to omega 3 supplementation impacted results but did not find a significant association between omega 3 supplementation and change in gut biomarkers. How specific populations of gut microbiota change in response to omega-3 supplementation remains incompletely understood and we were not able to quantify how supplementation impacted specific species of gut microbiota here.

This study has a number of strengths and limitations. It is one of few studies to evaluate the impact of a 12-week exercise program upon biomarkers of gut barrier permeability in a population with CAD. In addition, we were able to incorporate an objective measure of cardiorespiratory fitness assessed at baseline and study completion. Limitations include our small sample size and dependence upon blood-based biomarkers to assess intestinal permeability. In addition, we did not have fecal samples which would have allowed greater understanding of how gut microbiota composition was impacted by exercise. In conclusion, we found that an increase in cardiorespiratory fitness, as measured by VO_2peak_, during a 12-week exercise intervention was associated with a relative reduction in a biomarker of gut barrier integrity, FABP2. This provides increased understanding of how exercise may modulate gut barrier integrity in medical conditions associated with breakdown of the gut barrier. Findings should be replicated in larger sample sizes incorporating additional measures of intestinal permeability and assessing changes in composition of gut microbiota during exercise.
